# Time to take the gloves off: the use of radiation reduction gloves can greatly increase patient dose

**DOI:** 10.1120/jacmp.v15i6.5002

**Published:** 2014-11-08

**Authors:** Alexander S. Pasciak, A. Kyle Jones

**Affiliations:** ^1^ Department of Radiology University of Tennessee Graduate School of Medicine Knoxville TN USA; ^2^ Department of Imaging Physics University of Texas MD Anderson Cancer Center Houston TX USA

**Keywords:** radiation protection, radiation reduction gloves, fluoroscopy, tissue effects, occupational dose

## Abstract

Sterile radiation reduction gloves have been widely used over the past several decades in an effort to reduce hand doses during fluoroscopically guided procedures. While multiple studies have quantified the potential dose reduction to the fluoroscope operator from the use of such gloves, possible effects on the patient have not yet been quantified. The aim of this study was to examine the impact on patient dose when radiation reduction gloves are used. The impact on patient dose when using radiation reduction gloves in the field of view (FOV) was evaluated by measuring patient entrance surface dose rates (ESDR) using three C‐arm fluoroscopes for a range of patient sizes and different operating and magnification modes. Multiple measuring fields were used in combination with both peripheral and central glove placement. ESDR were measured with no glove in the FOV, with one radiation reduction glove, and with double gloves in the FOV, to replicate the actions of some fluoroscope operators. Compared to an ungloved hand, the use of a single radiation reduction glove in the measuring field resulted in up to a 2.8‐fold increase in ESDR. The use of double radiation reduction gloves resulted in up to a 4.9‐fold increase in ESDR. In both cases, the increase in ESDR was dependent on the size of the patient and on the operating and magnification modes used, and ranged from no increase up to the aforementioned maximum. When used in the FOV, and particularly within the measuring field, radiation reduction gloves can substantially increase ESDR. This increase in dose, when considered against the relatively small published reduction in dose to the operator's hands, may mean that the increased risks from the use of radiation reduction gloves outweigh the benefits. In any case, hands should not be placed in the FOV if not required by the goals of the procedure.

PACS number: 87.59.C‐

## I. INTRODUCTION

Sterile radiation reduction gloves have been widely used over the past several decades as a means to reduce operator extremity dose during fluoroscopically guided procedures. Radiation reduction gloves (RRG) are used primarily as a tool to attenuate scattered radiation to which the hands of the operator are exposed. Under conditions where the hands remain outside of the field of view (FOV), RRG may be useful to the fluoroscope operator by reducing extremity dose, specifically in lengthy cases with larger patients where the intensity of scattered radiation is higher. However, in some cases RRG are used to reduce dose to the hands from the primary beam when the operator's hands are placed in the FOV. While the hands are occasionally placed in the FOV during certain interventional radiology and cardiology procedures, this practice is more common and sometimes necessary to accomplish the goals of the procedure during open surgical procedures utilizing fluoroscopy, including many fluoroscopically guided orthopedic procedures.

Several United States patents describe possible compositions and manufacturing techniques for RRG.^1^, ^2^ The composition is typically polyvinyl chloride resin set with fine particles of tungsten or bismuth oxide,^1^, ^2^ although the use of lower atomic number absorbers has been suggested.^3^, ^4^ The typical material thickness of an RRG is less than 0.4 mm,^(5)^ with tactile sensation similar to wearing double surgical gloves. However, owing to the use of low material thickness and compositions that typically include more than 50% low atomic number binding material by volume,^(2)^ RRG are able to provide only moderate attenuation of X‐rays in the diagnostic energy range.

Experiments evaluating the protective value of RRG have been performed in actual clinical situations with ring dosimeters and simulated clinical conditions with phantoms and gas‐filled radiation detectors. Reported results for protective value vary widely, ranging from a reduction of hand dose by as little as 12%^(6)^ to as much as 75%;^(7)^ however, most studies report only a modest dose reduction.^6^, ^8^, ^9^, ^10^ In the end, multiple authors^6^, ^11^, ^12^, ^13^ have recommended against the use of RRG owing to the fact that they offer only modest dose reduction. Published recommendations of the National Council on Radiation Protection,^(14)^ the International Commission on Radiation Protection,^15^, ^16^ and guidelines of interventional radiology and cardiology professional societies^(17)^ have made similar recommendations, discouraging the use of RRG, particularly when the hand is placed in the FOV.

While the benefits of RRG to the wearer when the hands are placed in the FOV have been studied and shown to be small, only a few reports discuss the possible effects of the use of RRG on patient dose.^14^, ^15^, ^16^ However, these reports mention only that the potential for increased patient dose exists, but do not quantify this increase. The aim of this study was to quantify the impact on patient entrance surface dose rates (ESDR) when RRG are used in the FOV, compared to an ungloved hand.

## II. MATERIALS AND METHODS

The effect of RRG on ESDR was evaluated using three C‐arm fluoroscopes, one Axiom Artis flat‐panel fluoroscope, and Axiom Artis and AngioStar Plus image intensified systems (Siemens Medical Systems, Malvern, PA). The combined tissue attenuation of the patient and the fluoroscope operator's hand were simulated using 2.54 cm (1 inch) slabs of poly(methyl methacrylate) (PMMA) with total thickness ranging from 15 cm to 35 cm (6 inches to 14 inches). ESDR was measured on the Axiom Artis image intensified system using a 6 cc ionization chamber (model 10x6, Radcal Corp., Monrovia, CA). The ionization chamber was placed immediately below the phantom, which was elevated above the table slightly using spacers. ESDR was obtained from the air kerma rate $\left(K_{a}\right)$ measured using the ionization chamber with Eq. (1). Measurements on the Artis flat‐panel and AngioStar image‐intensifier fluoroscopes were performed with a solid‐state radiation dosimeter with lead backing (RaySafe Xi Platinum, Unfors RaySafe, Hopkinton, MA). Because this detector is radio‐opaque, it was placed at the periphery of the FOV, using the center circular measuring field of the fluoroscope such that the presence of the dosimeter did not affect the automatic dose‐rate control (ADRC) feedback. Measurements using the lead‐backed dosimeter were free from the influence of backscatter, and previously published backscatter factors as a function of kVp, added filtration, and X‐ray field size^(18)^ were applied after measurement using the technical factors reported by the fluoroscope:

(1) $${\text{ESDR}}_{\text{ic}}\left(\frac{\text{mGy}}{\text{min}}\right)=f\cdot K_{a}\left(\frac{\text{mGy}}{\text{min}}\right)$$

(2) $${\text{ESDR}}_{\text{ss}}\left(\frac{\text{mGy}}{\text{min}}\right)=f\cdot B\cdot K_{a}\left(\frac{\text{mGy}}{\text{min}}\right)$$

where ${\text{ESDR}}_{\text{ic}}$ was the ESDR for measurements performed using an ionization chamber, ${\text{ESDR}}_{\text{ss}}$ was the ESDR for measurements performed using the solid‐state dosimeter, *f* was the conversion factor from air kerma $\left(K_{a}\right)$ to tissue dose (equal to 1.06 mGy/mGy), and *B* was the backscatter factor.^(18)^ Backscatter factors from 1.36–1.5 were used depending on kVp, added filtration, and field size.

Boston Scientific ESP Radiation Reduction Gloves (Boston Scientific, Natick, MA) were attached to the center of a cardboard cutout with length and width matching the size of the PMMA slabs used to simulate tissue attenuation in order to maintain the RRG at the same position within the FOV for each measurement. The aim of this study was to examine the impact of wearing an RRG on patient dose. It is presupposed that the goals of the procedure dictate a necessity for the fluoroscope operator to place a hand in the FOV. Thus, the attenuation of the operator's ungloved hand can be considered as included in the PMMA absorber.

ESDR was measured on both Artis fluoroscopes for common fluoroscopic operating modes including normal fluoroscopy and high‐level fluoroscopy at both 7.5 pulses per second and 15 pulses per second. ESDR was measured in continuous mode on the AngioStar system. Measurements were performed using the 40, 28, 20, and 14 cm magnification modes on the image‐intensifier fluoroscopes, and using the 42, 32, 22, and 16 cm magnification modes on the flat‐panel fluoroscope. Additionally, ESDR was measured using acquisition mode on the Artis flat‐panel and AngioStar image‐intensifier fluoroscopes for the aforementioned magnification modes at frame rates of 2 and 3 frames per second. ESDR was measured with no RRG in the FOV, with a single RRG centered in the FOV (single‐glove technique), and with two RRG in the FOV (double‐glove technique) for all scenarios described above. The center circular measuring field was used for all measurements.

**Figure 1:**
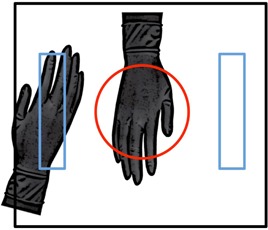
RRG location in relation to the visible field of view, indicated with a black rectangle. The left and right rectangular measuring fields are denoted with blue rectangles. The central circular measuring field is denoted in red. The positioning of the gloves relative to each measuring field is indicated.

The impact on ESDR of using different measuring fields was also evaluated, along with variations in the location of the RRG within the FOV of the flat‐panel fluoroscope. Measurements were made using central and left‐peripheral glove placement combined with use of the central circular, right rectangular, and adaptive^*^ measuring fields (Fig. 1). An attenuator thickness of 25.4 cm (10 inches) was used with the 42, 32, and 22 cm magnification modes for the central circular and adaptive measuring fields. Only the 42 and 32 cm magnification modes were used with the right rectangular measuring field, as it was not available at smaller FOV. Any object, including an ionization chamber, placed in the FOV will affect the air kerma rate when using the adaptive measuring field. Therefore, the installed kerma area product meter was used for these measurements following calibration to an external ionization chamber. The source‐to‐image distance was maintained at 120 cm for all measurements.

## III. RESULTS

Under many conditions, the use of either a single‐ or double‐glove technique resulted in a substantial increase in ESDR on the image‐intensifier fluoroscopes. The maximum increase in ESDR following introduction of a single RRG into the FOV was a factor of 2.6 and 2.8 for the Artis and AngioStar, respectively. Increases in ESDR exceeding a factor of 1.5 were found for all operating and magnification modes evaluated for attenuator thicknesses less than 20 cm. When using high magnification modes at large attenuator thicknesses, the increase in ESDR was minimal on both image intensified systems because the radiation output rate of the system reached the regulatory air kerma rate limit (88 mGy/min in normal fluoroscopy, 176 mGy/min in high‐level fluoroscopy) prior to placement of the RRG within the FOV.

**Figure 2:**
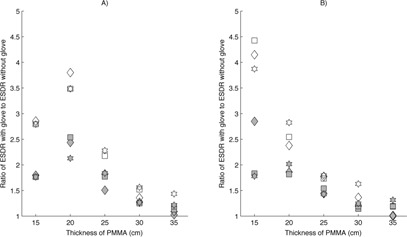
Relative increase in entrance surface dose rate (ESDR) when using a single‐glove (filled symbols) or double‐glove technique (open symbols) during fluoroscopy on a Siemens Axiom Artis image‐intensifier fluoroscope. A value of 1.0 indicates no change when using an RRG. ♦ is fluoroscopy at 7.5 pulses per second (pps), ▪ is fluoroscopy at 15 pps, ⋆ is high level fluoroscopy at 15 pps: (a) 40 cm magnification mode; (b) 28 cm magnification mode.

**Figure 3:**
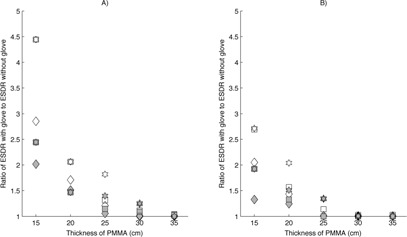
Relative increase in entrance surface dose rate (ESDR) when using a single‐glove (filled symbols) or double‐glove technique (open symbols) during fluoroscopy on a Siemens Axiom Artis image‐intensifier fluoroscope. A value of 1.0 indicates no change when using an RRG. ♦ is fluoroscopy at 7.5 pulses per second (pps), ▪ is fluoroscopy at 15 pps, ⋆ is high level fluoroscopy at 15 pps: (a) 20 cm magnification mode; (b) 14 cm magnification mode.

**Figure 4:**
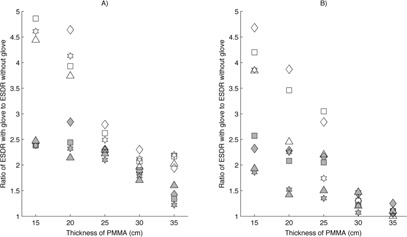
Relative increase in entrance surface dose rate (ESDR) when using a single‐glove (filled symbols) or double‐glove technique (open symbols) during fluoroscopy on a Siemens AngioStar image‐intensifier fluoroscope. A value of 1.0 indicates no change when using an RRG. (a) 40 and 28 cm magnification modes: ♦ is continuous fluoroscopy using a 40 cm magnification mode, ▪ is acquisition imaging at 3 frames per second (fps) using a 40 cm magnification mode, ⋆ is continuous fluoroscopy using a 28 cm magnification mode, and ▴ is acquisition imaging at 3 fps using a 28 cm magnification mode. (b) 20 and 14 cm magnification modes: ♦ is continuous fluoroscopy using a 20 cm magnification mode, ▪ is acquisition imaging at 3 fps using a 20 cm magnification mode, ⋆ is continuous fluoroscopy using a 14 cm magnification mode, and ▴ is acquisition imaging at 3 fps using a 14 cm magnification mode.

Similar results were noted on the image‐intensifier fluoroscopes when using the double‐glove technique, except that the maximum increase in ESDR was higher. A maximum increase in ESDR of a factor of 4.9 was noted for an attenuator thickness of 15 cm using the 28 cm magnification mode on the AngioStar plus system. Because the presence of two RRG in the FOV more quickly drove the ADRC to increase the radiation output to levels approaching the regulatory air kerma rate limit, the relative increase in ESDR was reduced quickly as attenuator thicknesses reached 25 or more cm of PMMA. Figures 2, 3, 4 illustrate the increase in ESDR for single‐ and double‐glove techniques.

**Figure 5:**
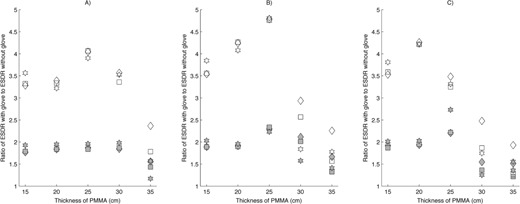
Relative increase in entrance surface dose rate (ESDR) when using a single‐glove (filled symbol) or double‐glove technique (open symbol) during fluoroscopy on a Siemens Axiom Artis flat‐panel fluoroscope. A value of 1.0 indicates no change when using an RRG. ♦ is fluoroscopy at 7.5 pulses per second (pps), ▪ is fluoroscopy at 15 pps, ⋆ is high level fluoroscopy at 15 pps: (a) 42 cm magnification mode; (b) 32 cm magnification mode; (c) 22 cm magnification mode.

The response of the flat‐panel fluoroscope when using a single‐glove technique was similar in magnitude to that of the image‐intensifier fluoroscope, but with less variation with changes in attenuator thickness. The average increase in ESDR at the 42 cm magnification mode was a factor of 2.0 for all operating modes and attenuator thicknesses less than 30.5 cm. The use of higher magnification modes resulted in similar increases in ESDR, averaging a factor of 2.1 for attenuator thicknesses less than 30.5 cm; however, the results had more variation than was observed using the 42 cm mode. These relationships are illustrated in Fig. 5.

The use of a double‐glove technique with the flat‐panel fluoroscope resulted in substantial increases in ESDR, peaking at a factor of 4.8, but exhibiting quick falloff with increasing attenuator thickness owing to the regulatory air kerma rate limits. Across all magnification and operating modes evaluated, the average increase in ESDR was a factor of 3.8 for attenuator thicknesses less than 25.4 cm of PMMA. Figure 4 illustrates the increase in ESDR on a flat‐panel fluoroscope.

**Figure 6:**
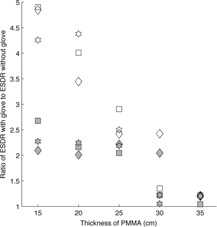
Relative increase in entrance surface dose rate (ESDR) when using a single‐glove (filled symbol) or double‐glove technique (open symbol) during acquisition at 3 frames per second (fps) on a Siemens Axiom Artis flat‐panel fluoroscope. A value of 1.0 indicates no change when using an RRG. ♦ is the 42 cm magnification mode, ▪ is the 32 cm magnification mode, ⋆ is the 22 cm magnification mode.

Figure 6 illustrates the effect of single‐ and double‐glove techniques on ESDR for different magnification modes when the flat‐panel fluoroscope was operated in acquisition mode. The increase in ESDR observed during acquisition was similar to the increase observed during fluoroscopy. When a single‐glove technique was used, an average increase in ESDR of a factor of 2.2 was seen for attenuator thicknesses less than 25.4 cm across all magnification modes. Similar to fluoroscopy, the rate of increase slowed at larger attenuator thicknesses; however, in this case, it was a consequence of the radiation output limitations of the X‐ray tube and generator rather than the regulatory air kerma rate limits. The use of a double‐glove technique resulted in increases approaching a factor of 5.0 compared to not using RRG, with a quick falloff as attenuator thickness increased owing to physical limitations on radiation output rate. When the AngioStar image‐intensifier fluoroscope was operated in acquisition mode, observed increases in ESDR were similar to the Artis flat‐panel system for both single‐ and double‐glove techniques.

Once again, the relative increase in ESDR decreased quickly as attenuator thickness increased owing to X‐ray tube output limitations. These data are shown in Fig. 4.

**Figure 7:**
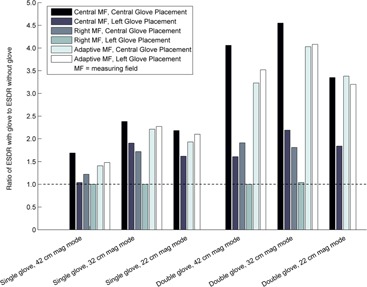
Relative increase in entrance surface dose rate (ESDR) when using a single‐ or double‐glove technique with a 25.4 cm attenuator using different measuring fields and varying glove placement within the field of view. A value of 1.0 indicates no change when using an RRG.

When using the central circular measuring field, the use of an RRG within the FOV resulted in a large increase in ESDR. Central placement of the glove within the FOV when using the central measuring field resulted in the greatest increase in ESDR, as the glove fully obscured the measuring field regardless of the magnification mode used. Peripheral placement of the glove in the FOV when using the central measuring field resulted in a negligible increase in ESDR for the 42 cm magnification mode when a single‐glove technique was used. However, increases in ESDR were observed when using higher magnification modes or a double‐glove technique. Central placement of the glove within the FOV when using the right rectangular measuring field resulted in an increase in ESDR similar to peripheral glove placement when using the central measuring field. Using either a single‐ or double‐glove technique did not affect the ESDR when left peripheral placement of the glove in the FOV was used with the right rectangular measuring field, regardless of the magnification mode used. Use of the adaptive measuring field resulted in a substantial increase in ESDR, almost as great as the increase in ESDR observed when using the central glove placement within the FOV with the central measuring field. Negligible differences in ESDR were observed as the position of the glove in the FOV varied when using the adaptive measuring field. Figure 7 summarizes the impact of measuring field and glove location in the FOV on ESDR when using both single‐ and double‐glove techniques.

## IV. DISCUSSION

Except for the specific case of peripheral glove location outside of the measuring field, the use of either a single‐ or double‐glove technique resulted in an increase in ESDR in scenarios where attenuator thicknesses were small (<20cm). In several cases, the increase was large, exceeding a factor of 2 for a single‐glove technique and a factor of 4 for a double‐glove technique. As shown in Figs. 2, 3, 4, 5, 6, an increase in ESDR was observed regardless of the mode of operation, magnification mode, or pulse rate. When located within the measuring field, RRG not only drive the ADRC to increase the radiation output rate, they also trigger other changes that tend to increase the ESDR. Depending on the operational logic of the ADRC,^19^, ^20^ an RRG may cause a fluoroscope to remove added filtration, decreasing beam quality and further increasing the ESDR, or may cause the ADRC to increase the kVp, increasing the backscatter factor and, therefore, the ESDR. It is likely that these factors contributed to the increases in ESDR shown in Figs. 2, 3, 4, 5, 6, 7.

The effect of using a single‐ or double‐glove technique during acquisition imaging on a flat‐panel fluoroscope was similar to the effect observed during fluoroscopy, including falloff in the rate of increase of ESDR at large attenuator thicknesses. As ESDR is 40–80 times higher during acquisition imaging compared to fluoroscopy, the use of either a single‐ or double‐glove technique can be particularly detrimental. Because RRG provide only a modest dose reduction to the hand,^6^, ^8^, ^9^, ^10^ placement of a gloved hand in the FOV during acquisition imaging will still result in a substantial hand dose. More importantly, the results of this study demonstrate that the use of a single‐ or double‐glove technique may result in a large increase in ESDR. Because modern fluoroscopes are capable of producing ESDR in excess of 2 Gy/min, the use of even a single‐glove technique has the potential to greatly increase peak skin dose.

The location of an RRG when placed in the FOV also influenced the ESDR, with the impact depending on the measuring field used. Increases in ESDR can be minimized by keeping the RRG out of the measuring field. Commensurate with the goals of the procedure, a different measuring field, if available, opposite the location of the glove can be used with little or no resulting increase in ESDR. Adaptive (i.e., histogram‐based) measuring fields should not be used with RRG, as this practice will always result in an increase in ESDR.

A limitation of this study was that the effect of RRG on ESDR was evaluated on only three C‐arm fluoroscopes, using RRG from a single manufacturer. ADRC systems on fluoroscopes from other manufacturers, and even those on different fluoroscopes from the same manufacturer, may follow different operation logic, with different impacts on ESDR. However, while different fluoroscopes will behave differently, ESDR will increase when an RRG is placed in the FOV and, in particular, when placed within the measuring field.

## V. CONCLUSIONS

The hands of the fluoroscope operator should only be placed in the FOV when required by the goals of the procedure, whether a RRG is used or not. Current national and international guidelines^14^, ^15^, ^17^ recommend against the use of RRG when the hands are placed in the FOV, primarily owing to limited hand protection they provide. While these guidelines also mention that the use of RRG in the FOV may result in an increase in patient dose, this work quantified the expected increase. When used in the FOV, RRG can substantially increase ESDR. The increase observed in this study ranged from 0% to almost 500%, depending on factors such as patient size and fluoroscopic technique factors. These potential increases in ESDR should motivate the fluoroscope operator to carefully consider risks and benefits of radiation reduction gloves before using them during procedures where the hands may enter the FOV. However, it should be emphasized that the use of RRG to reduce the exposure of the hands to scattered radiation when the hands remain outside the FOV is not contraindicated.
